# An efficient clustering algorithm for partitioning Y-short tandem repeats data

**DOI:** 10.1186/1756-0500-5-557

**Published:** 2012-10-06

**Authors:** Ali Seman, Zainab Abu Bakar, Mohamed Nizam Isa

**Affiliations:** 1 Center for Computer Sciences, Faculty of Computer and Mathematical Sciences, Universiti Teknologi MARA (UiTM), 40450 Shah Alam, Selangor, Malaysia; 2 Medical Faculty, Masterskill University College of Health Sciences, No. 6, Jalan Lembah, Bandar Seri Alam, 81750, Johor Bahru, Johor, Malaysia

**Keywords:** Algorithms, Bioinformatics, Clustering, Optimization, Data mining

## Abstract

**Background:**

Y-Short Tandem Repeats (Y-STR) data consist of many similar and almost similar objects. This characteristic of Y-STR data causes two problems with partitioning: non-unique centroids and local minima problems. As a result, the existing partitioning algorithms produce poor clustering results.

**Results:**

Our new algorithm, called *k*-Approximate Modal Haplotypes (*k*-AMH), obtains the highest clustering accuracy scores for five out of six datasets, and produces an equal performance for the remaining dataset. Furthermore, clustering accuracy scores of 100% are achieved for two of the datasets. The *k*-AMH algorithm records the highest mean accuracy score of 0.93 overall, compared to that of other algorithms: *k*-Population (0.91), *k*-Modes-RVF (0.81), New Fuzzy *k*-Modes (0.80), *k*-Modes (0.76), *k*-Modes-Hybrid 1 (0.76), *k*-Modes-Hybrid 2 (0.75), Fuzzy *k*-Modes (0.74), and *k*-Modes-UAVM (0.70).

**Conclusions:**

The partitioning performance of the *k*-AMH algorithm for Y-STR data is superior to that of other algorithms, owing to its ability to solve the non-unique centroids and local minima problems. Our algorithm is also efficient in terms of time complexity, which is recorded as *O*(*km*(*n-k*)) and considered to be linear.

## Background

Y-Short Tandem Repeats (Y-STR) data represent the number of times an STR motif repeats on the Y-chromosome. It is often called the allele value of a marker. For example, if there are eight allele values for the DYS391 marker, the STR would look like the following fragments: [TCTA] [TCTA] [TCTA] [TCTA] [TCTA] [TCTA] [TCTA] [TCTA]. The number of tandem repeats has effectively been used to characterize and differentiate between two people.

In modern kinship analyses, the Y-STR is very useful for distinguishing lineages and providing information about lineage relationships [1]. Many areas of study, including genetic genealogy, forensic genetics, anthropological genetics, and medical genetics, have taken advantage of the Y-STR method. For example, it has been used to trace a similar group of Y-surname projects to support traditional genealogical studies, e.g., [2-4]. Further, in forensic genetics, the Y-STR is one of the primary concerns in human identification for sexual assault cases [5], paternity testing [6], missing persons [7], human migration patterns [8], and the reexamination of ancient cases [9].

From a clustering perspective, the goal of partitioning Y-STR data is to group a set of Y-STR objects into clusters that represent similar genetic distances. The genetic distance of two Y-STR objects is based on the mismatch results from comparing the Y-STR objects and their modal haplotypes. For Y-surname applications, if two people share 0, 1, 2, and 3 allele value mismatches for each marker, they are considered to be the most familially related. Furthermore, for Y-haplogroup applications, the number of mismatches is variant and greater than that typically found in Y-surname applications. This is because the haplogroup application is based on larger family groups branched out from the same ancestor, covering certain geographical areas and ethnicities throughout the world. The established Y-DNA haplogroups named by the letters A to T, with further subdivisions using numbers and lower case letters, are now available for reference (see [10] and [11] for details).

Efforts to group Y-STR data based on genetic distances have recently been reported. For example, Schlecht *et al*. [12] used machine learning techniques to classify Y-STR fragments into related groups. Furthermore, Seman *et al*. [13-19] used partitional clustering techniques to group Y-STR data by the number of repeats, a method used in genetic genealogy applications. In this study, we continue efforts to partition the Y-STR data based on the partitional clustering approaches carried out in [13-19]. Recently, we have also evaluated eight partitional clustering algorithms over six Y-STR datasets [19]. As a result, we found that there is scope to propose a new partitioning algorithm to improve the overall clustering results for the same datasets.

A new partitioning algorithm is required to handle the characteristics of Y-STR data, thus producing better clustering results. Y-STR data are slightly unique compared to the common categorical data used in [20-25]. The Y-STR data contain a higher degree of similarity of Y-STR objects in their intra-classes and inter-classes. (Note that the degree of similarity is based on the mismatch results when comparing the objects and their modal haplotypes.) For example, many Y-STR surname objects are found to be similar (zero mismatches) and almost similar (1, 2, and 3 mismatches) in their intra-classes. In some cases, the mismatch values of inter-class objects are not obviously far apart. Y-STR haplogroup data contain similar, almost similar, and also quite distant objects. Occasionally, the Y-STR haplogroup data may include sub-classes that are sparse in their intra-classes.

### Partitional clustering algorithms

Classically, clustering has been divided into hierarchical and partitional methods. The main difference between the two is that the hierarchical method breaks the data up into hierarchical clusters, whereas the partitional method divides the data into mutually disjoint partitions. The pillar of the partitional algorithms is the *k*-Means algorithm [26], introduced almost four decades ago. As a consequence, the *k*-Means paradigm has been extended to various versions, including the *k*-Modes algorithm [25] for categorical data.

The *k*-Modes algorithm owes its existence to the ineffectiveness of the *k*-Means algorithm for handling categorical data. Ralambondrainy [27] attempted to rectify this using a hybrid numeric–symbolic method based on the binary characters 0 and 1. However, this approach suffered from an unacceptable computational cost, particularly when the categorical attributes had many categories. Since then, a variety of *k*-Modes-type algorithms have been introduced, such as *k*-Modes with new dissimilarity measures [21,22], *k*-Population [23], and a new Fuzzy *k*-Modes [20].

Partitional algorithms use an objective function in their optimization process, and the determination of this function was described as the *P* problem by Bobrowski and Bezdek [28] and Salim and Ismail [29]. When he proposed the *k*-Modes clustering algorithm, Huang [25] split *P* into *P*_*1*_ and *P*_*2*_. *P*_*1*_ denotes the minimization problem of obtaining values for the partition matrix *w*_*li*_ of 0 or 1 (for the hard clustering approach) or 0 to 1 (for the fuzzy clustering approach); see Eq. (1b) as an example. Furthermore, *P*_*2*_ denotes the minimization problem of obtaining the value that occurs most often (or the mode of a categorical data set) to represent the center of a cluster (often called the centroid). The minimization of *P*_*2*_ by obtaining the appropriate mode essentially causes the minimization of problem *P*_*2*_, and vice versa. As an example of the optimization process for problem *P* in the Fuzzy *k*-Modes algorithm, we wish to solve Eq. (1) subject to Eqs. (1a), (1b), and (1c).

(1) $$P\left(W,Z\right)=\sum_{l=1}^{k}\sum_{i=1}^{n}w_{\mathrm{li}}^{\propto }d\left(X_{i},Z_{l}\right)$$

subject to:

(1a) $$\sum_{l=1}^{k}w_{\mathrm{li}}=1,1\leq i\leq n\text{,}$$

(1b) $$w_{\mathrm{li}}\in \left[0,1\right],1\leq i\leq n,1\leq l\leq k$$

And

(1c) $$0<\sum_{i=1}^{n}w_{\mathrm{li}}<n,1\leq l\leq k$$

where:

• *w*_*li*_ is a (*k* × *n*) partition matrix that denotes the degree of membership of object *i* in the *l*th cluster that contains a value of 0 to 1,

• *k (≤ n)* is a known number of clusters,

• *Z* is the centroid such that *[Z*_*1*_*, Z*_*2*_*,…,Z*_*k*_*] ∈ R*^*mk*^,

• α [1, ∞) is a weighting exponent,

• *d*(*X*_*i*_, *Z*_*l*_) is the distance measure between the object *X*_*i*_ and the centroid *Z*_*l*_, as described in Eqs. (2) and (2a).

(2) $$d\left(x,z\right)=\sum_{j=1}^{n}\delta \left(x_{j},z_{j}\right)$$

where:

(2a) $$\delta \left(x_{j},z_{j}\right)=\{\begin{array}{c} 0,x_{j}=z_{j}\\ 1,x_{j}\neq z_{j} \end{array}$$

Huang and Ng [24] described the optimization process of *P*_*1*_ and *P*_*2*_ as follows:

• Problem *P*_*1*_: Fix *Z* = $\hat{Z}$ and solve the reduced problem *P(W,*$\hat{Z}$*)* as in Eq. (3). This process obtains the minimized values of 0–1 of the partition matrix *w*_*li*_.

(3) wli={1,IfXi=Z^l0,IfXi=Z^h,h≠l1∑h=1kdXi,Z^ldXi,Z^h1α−1,IfXi≠Z^l,andXi≠X^h,1≤h≤k

• Problem *P*_*2*_: Fix *W* = *Ŵ* and solve the reduced problem *P(Ŵ, Z)* as in Eq. (4) subject to Eq. (4a). This process obtains the most frequent attributes, or the modes, which give the centroids.

(4) $$Z_{\mathrm{li}}=a_{j}^{\left(p\right)}\in \text{DOM}\left(A_{j}\right)$$

where:

(4a) $$\sum_{i,x_{i,j=a_{j}^{\left(p\right)}}}w_{\mathrm{li}}^{\propto }\;\geq \;\sum_{i,x_{i,j=a_{j}^{\left(t\right)}}}w_{\mathrm{li}}^{\propto }\forall l,1\;\leq \;t\;\leq \;n_{j,}\;1\;\leq \;\leq \;m$$

and α ∈ [1, ∞) is a weighting exponent.

### Problem of partitioning Y-STR data

Due to the characteristics of Y-STR data, there are two optimization problems for existing partitional algorithms: non-unique centroids and local minima problems. These two problems are caused by the drawback of the modes mechanism of determining the centroids. Non-unique centroids would result in empty clusters, whereas the local minima problem leads to poorer clustering results. Both problems are a result of the obtained centroids, which are not sufficient to represent their classes.

Therefore, problems will occur for the following two cases:

i)The total number of objects in a dataset is small while the number of classes is large. To illustrate this case, consider the following example.

**Figure 1:**
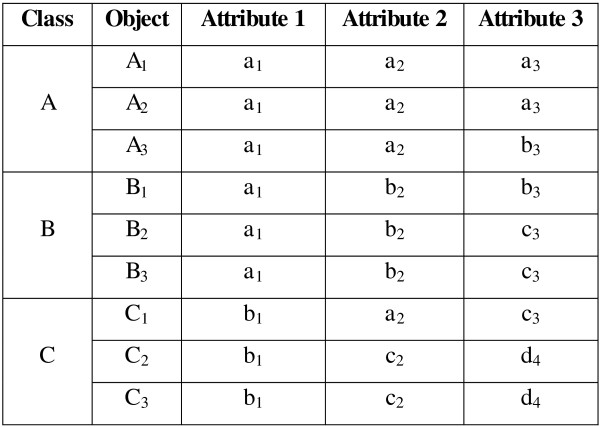
**Artificial Example 1. **An example of higher degree of similarity between objects.

**Figure 2:**
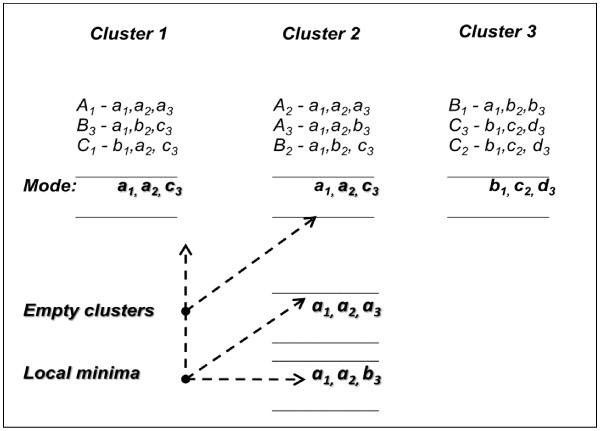
**The dominant attributes form centroid 1 (*a***_***1***_***, a***_***2***_***, c***_***3***_**)*, *centroid 2 (*a***_***1***_***, a***_***2***_***, c***_***3***_**) and centroid 3 (*b***_***1***_***, c***_***2***_***, d***_***3***_**). **In this case, there are possibilities that each cluster is formed by the dominant attributes, e.g. attribute *a*_*1*_, *a*_*2 *_and *c*_*3*. _This scenario of non-unique centroids would result in empty clusters; otherwise the centroids would lead to local a minima problem and produce poorer clustering results.

*Example I:* Figure 1 shows an artificial example of a dataset consisting of nine objects in three classes: Class *A* = {*A*_*1*_*, A*_*2*_*, A*_*3*_}, Class *B* = {*B*_*1*_*, B*_*2*_*, B*_*3*_}, and Class *C* = {*C*_*1*_*, C*_*2*_*, C*_*3*_}. Each object is composed of three attributes, represented in lower case; e.g., for object *A*_*1*_, the attributes are *a*_*1*_, *a*_*2*_, and *a*_*3*_. The dataset is considered to have a higher degree of similarity between objects in intra-classes, while the number of objects is small and number of classes is large. Thus, the appropriate modes for representing the classes are: Class *A* – [*a*_*1*_*, a*_*2*_*, a*_*3*_], lass *B* – [*a*_*1*_*, b*_*2*_*, c*_*3*_], and Class *C* – [*b*_*1*_*, c*_*2*_*, d*_*4*_]. However, attribute *a*_*1*_ in DOMAIN (*A*_*1*_), *a*_*2*_ in DOMAIN (*A*_*2*_), and *c*_*3*_ in DOMAIN (*A*_*3*_) are too dominant, and would therefore dominate the process of updating *P*_*2*_. Figure 2 shows the possibility that each cluster is formed by the dominant attributes.

As a result, the mode that consists of [*a*_*1*_*, a*_*2*_*, c*_*3*_] would be obtained twice. Thus, *P*_*2*_ would not be minimized due to this non-unique centroid. Another possibility is that the two modes are different, but are not distinctive enough to represent their clusters, such as modes [*a*_*1*_*, a*_*2*_*, a*_*3*_] or [*a*_*1*_*, a*_*2*_*, b*_*3*_] for Cluster 2. As a consequence, this case would fall into a local minima problem.

ii)An extreme distribution of objects in a class. To illustrate this case, consider the following example.

**Figure 3:**
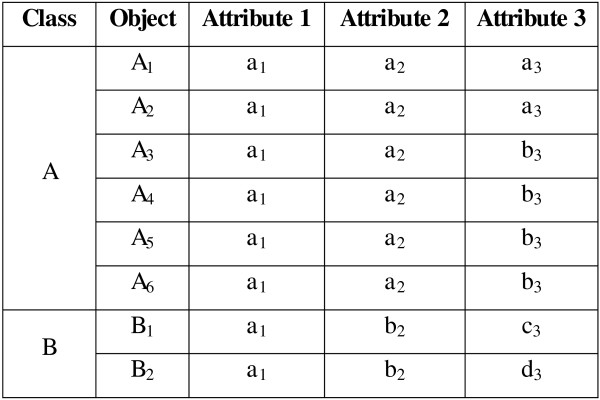
**Artificial Example 2. **An example of the extreme distribution of objects in a class.

**Figure 4:**
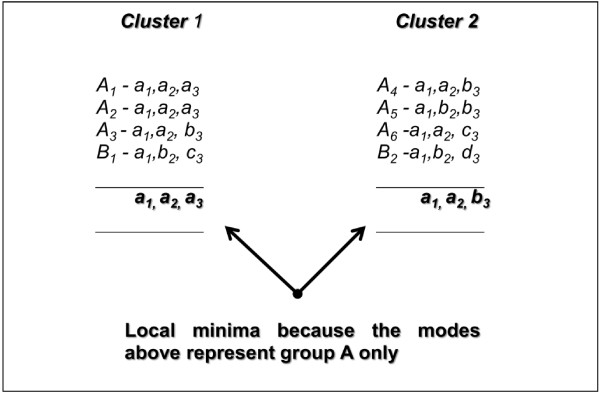
**The extreme distribution of objects *A *forms centroid 1 (*a***_***1***_**, *a***_***2***_**, *a***_***3***_**) and centroid 2 (*a***_***1***_**, *a***_***2***_**, *b***_***3***_**). **In this case, the objects in Class A are equally distributed into clusters 1 and 2. Therefore, the obtained centroids are not sufficient to represent their classes.

*Example II:* Figure 3 shows a dataset consisting of eight objects in two classes: Class *A* = {*A*_*1*_*, A*_*2*_*, A*_*3*_*, A*_*4*_*, A*_*5*_*, A*_*6*_} and Class *B* = {*B*_*1*_*, B*_*2*_}. Each object consists of three attributes, again represented in lower case. The appropriate modes to represent the classes are: Class *A* – [*a*_*1*_*, a*_*2*_*, b*_*3*_] and Class *B* – [*a*_*1*_*, b*_*2*_*, c*_*3*_] or [*a*_*1*_*, b*_*2*_*, d*_*3*_]. The distribution of objects in Class *A* is considerably larger than in Class *B*, covering approximately 75% of the total set of objects. This characteristic of the data is found to be problematic for *P*_*2*_, particularly for the fuzzy approach. The problem is actually caused by the initial centroid selection. Figure 4 shows the objects in Class *A* would be equally distributed into clusters 1 and 2.

As a result, object *A* becomes dominant in both clusters, and so the obtained modes might be represented solely by objects in Class *A*, e.g., [*a*_*1*_*, a*_*2*_*, a*_*3*_] and [*a*_*1*_*, a*_*2*_*, b*_*3*_].

The above situations cause *P* not to be fully optimized, thus producing poor clustering results. Therefore, a new algorithm with a new concept of *P*_*2*_ is proposed in order to overcome these problems and improve the clustering accuracy results of Y-STR data.

## Methods

### The center of a cluster

The mode mechanism for the center of a cluster (problem *P*_*2*_) is not appropriate for handling the characteristics of Y-STR data, and therefore, it cannot be used as a mechanism to represent the center of a cluster (centroid). Instead, the center of Y-STR data should be the modal haplotypes, which are required to calculate the distance of Y-STR objects. The distance between a Y-STR object and its modal haplotype can be formalized as in Eq. (5) subject to Eq. (5a).

(5) $$d_{\text{ystr}}\left(X,H\right)\;=\;\sum_{j=1}^{m}\left(x_{j},h_{j}\right)$$

subject to:

(5a) $$y\left(x_{j},h_{j}\right)\;=\;\{\begin{array}{c} 0,x_{j}=\;h_{j}\\ 1,x_{j}\neq \;h_{j} \end{array}$$

where *m* is the number of markers.

The modal haplotype is controlled by groups of objects that are similar or almost similar in Y-STR data. The similar and almost similar objects have a lower distance, or a higher degree of membership values in a fuzzy sense. Thus, these two groups are considerably the most dominant objects required to find the Approximate Modal Haplotype. Consider four objects *x*_*1*_*, x*_*2*_*, x*_*3*_, and *x*_*4*_ and two clusters *c*_*1*_ and *c*_*2*_. The membership value for each object and its cluster are as shown in Table 1, whereby objects *x*_*1*_ and *x*_*3*_ have a 100% chance of being the most dominant object in cluster *c*_*1*_, but only a 50% chance of being the dominant object in cluster *c*_*2*_, and so on. A dominant weighting value of 1.0 is given to any dominant object and a weight of 0.5 is given to the remaining objects.

**Table 1:** Example of dominant objects

**Objects**	**Membership Values**		**Probability of being the dominant object in the cluster**	
	***c***_***1***_	***c***_***2***_	***c***_***1***_	***c***_***2***_
*x*_*1*_	**0.7**	0.3	100% (1.0)	50% (0.5)
*x*_*2*_	0.4	**0.6**	50% (0.5)	100% (1.0)
*x*_*3*_	**0.6**	0.4	100% (1.0)	50% (0.5)
*x*_*4*_	0.3	**0.7**	50% (0.5)	100% (1.0)

### The *k*-AMH algorithm

Let *X* ={*X*_*1*_*, X*_*2*_*,…, X*_*n*_} be a set of *n* Y-STR objects and *A* ={*A*_*1*_*,A*_*2*_*,…, A*_*m*_} be a set of markers (attributes) of a Y-STR object. Let *H* = {*H*_*1*_*, H*_*2*_*,.,H*_*k*_} ∈ *X* be the set of Approximate Modal Haplotypes for *k* clusters. Suppose *k* is known a priori. Let *H*_*l*_ be the Approximate Modal Haplotype, represented as [*h*_*l,1*_*, h*_*l,2*_*,…,h*_*l,m*_], and therefore, *H*_*l,j*_ = *X*_*i,j*_ for 1*≤ j ≤ m and* 1*≤ i ≤ n*. The objective of the algorithm is to partition the categorical objects *X* into *k* clusters. Thus, the *H*_*l*_ can be replaced by *X*_*i*_ until *n* provided they satisfy the condition described in Eq. (6).

(6) $$P{\left(Á\right)}^{s}>\;P{\left(Á\right)}^{t},s\;\neq \;t;\;\forall t,\;1\;\leq \;t\;\leq \;\left(n-k\right)\text{.}$$

Here, *P(Á)* is the cost function described in Eq. (7), which is subject to Eqs. (7a), (8), (8a), (8b), (9), (9a), (9b), and (9c).

(7) $$P\left(Á\right)\;=\;\sum_{l=1}^{k}\sum_{i=1}^{n}{Á}_{\mathrm{li}}$$

subject to:

(7a) $${Á}_{\mathrm{li}}=W_{\mathrm{li}}^{\propto }D_{\mathrm{li}}$$

• *W*_*li*_^∝^ is a (*k* × *n*) partition matrix that denotes the degree of membership of Y-STR object *i* in the *l*th cluster that contains a value of 0 to 1 as described in Eq. (8), subject to Eqs. (8a) and (8b).

(8) Wli∝={1,If,Xi=Hi0,If,Xi=Hz,z≠l1∑z=1kdystrXi,HldystrXi,Hz1∝−1,IfHi≠XjandXi≠Hz,1≤z≤k∝

• subject to:

(8a) $$w_{\mathrm{li}}^{\propto }\;\in \;\left[0,1\right],\;1\;\leq \;i\;\leq \;n,\;1\;\leq \;l\;\leq \;k\text{,}$$

and

(8b) $$0\;<\;\sum_{i=1}^{n}w_{\mathrm{li}}^{\propto }\;<\;n,\;1\;\leq \;l\;\leq \;k$$

where,

• *k (≤ n)* is a known number of clusters.

• *H* is the Approximate Modal Haplotype (centroid) such that *[H*_*1*_*, H*_*2*_*,…,H*_*k*_*] ∈ X*.

• α ∈ [1, ∞) is a weighting exponent and used to increase the precision of the membership degrees. Note that this alpha is typical based on 1.1 until 2.0 as introduced by Huang and Ng [24].

• *d*_*ystr*_(*X*_*i*,_*H*_*l*_) is the distance measure between the Y-STR object *X*_*i*_ and the Approximate Modal Haplotype *H*_*l*_ as described in Eq. (5) and subject to Eq.(5a).

• *D*_*li*_ is another (*k* × *n*) partition matrix which contains a dominant weighting value of 1.0 or 0.5, as explained above (See Table 1). The dominant weighting values are based on the value of *W*_*li*_^∝^ above. *D*_*li*_ is described in Eq. (9), subject to Eqs. (9a), (9b), and (9c).

(9) $$d_{\mathrm{li}}\;=\;\{\begin{array}{c} 1.0,\;\text{if}\;w_{\mathrm{li}}^{\propto }\;=\;{\text{max}}^{w_{\mathrm{li}}^{\propto },1\;\leq \;l\;\leq \;k}\\ 0.5,\;\text{otherwise} \end{array}$$

subject to:

(9a) $$d_{\mathrm{li}}\;\in \;\left\{1,0.5\right\},\;1\;\leq \;i\;\leq \;n,\;1\;\leq \;l\;k$$

(9b) $$1.5\;\leq \;\sum_{l=1}^{k}d_{\mathrm{li}}\;\leq \;k,\;1\;\leq \;i\;\leq \;n$$

(9c) $$1.5\;\leq \;\sum_{l=1}^{k}d_{\mathrm{li}}\;\leq \;n,\;1\;\leq \;i\;\leq \;k$$

The basic idea of the *k*-AMH algorithm is to find *k* clusters in *n* objects by first randomly selecting an object to be the Approximate Modal Haplotype *h* for each cluster. The next step is to iteratively replace the objects *x* one-by-one towards the Approximate Modal Haplotype *h*. The replacement is based on Eq. (6) if the cost function as described in Eq. (7) and subject to (7a), (8), (8a), (8b), (9), (9a), (9b) and, (9c) is maximized. Thus, the differences between the *k*-AMH algorithm and the other *k*-Mode-type algorithms are as follows.

i. The objects (the data themselves) are used as the centroids instead of modes. Since the distance of Y-STR objects is measured by comparing the objects and their modal haplotypes, we need to approximately find the objects that can represent the modal haplotypes. In finding the final Approximate Modal Haplotype for a particular group (cluster), each object needs to be tested one-by-one and replaced on a maximization of a cost function.

ii. A maximization process of the cost function is required instead of minimizing it as in the *k*-mode-type algorithms.

A detailed description of the *k*-AMH algorithm is given below.

Step 1 – Select *k* initial objects randomly as Approximate Modal Haplotype (centroids)*.* E.g. if *k* = 4, then choose randomly 4 objects as the initial Approximate Modal Haplotype.

Step 2 – Calculate distance *d*_*ystr*_(*X*_*i*,_*H*_*l*_) according to Eq. (5) and subject to (5a).

Step 3 – Calculate partition matrix *w*_*li*_^∝^ according to Eq. (8), subject to Eqs. (8a) and (8b). Note that the *w*_*li*_^∝^ is based on the distance calculated in Step 2.

Step 4 – Assign a weighting dominant of 1.0 or 0.5 for partition matrix *D*_*li*_ according to Eqs. (9), (9a), (9b) and (9c).

Step 5 – Calculate cost function *P*(*Á*) based on *W*_*li*_^∝^*D*_*li*_ according to Eqs (7) and (7a).

Step 6 – Test for each initial modal haplotype by the other objects one-by-one. If current cost function is greater than previous cost function according to Eq. (6), then replace it.

Step 7 – Repeat Step 2 until Step 6 for each *x* and *h*

Step 8 – Once the final Approximate Modal Haplotypes are obtained for all clusters, assign the objects to their corresponding crisp clusters *C*_*li*_ according to Eq. (10).

(10) $$C_{\mathrm{li}}\;=\;\{\begin{array}{c} 1,\;\text{if}\;l\;=\;\text{arg}\;\text{max}\;w_{\mathrm{li}}^{\propto }\;,\;1\;\leq \;j\;\leq \;c\\ 0,\text{otherwise} \end{array}$$

Furthermore, the implementation of the steps above of the algorithm is formalized in the form of pseudo-code as follows.

**INPUT:** Dataset, ***X***, the number of cluster, ***k****,* the number of dimensional, ***d*** and the fuzziness index,

**OUTPUT**: A set of clusters, ***k***

01: Select *H*_***l***_ randomly from ***X****such that 1≤l≤ k*

02: for each *H*_***l***_ an Approximate Modal Haplotype do

03: for each *X*_*i*_ do

04: Calculate *P*(*À*)  =   ∑ _*l* = 1_^*k*^ ∑ _*i* = 1_^*n*^*À*_*li*_

05: if *P*(*À*)  =   ∑ _*l* = 1_^*k*^ ∑ _*i* = 1_^*n*^*À*_*li*_ is maximized, then

06: Replace *H*_***l***_ by *X*_***i***_

07: end if end for

09: end for

10: Assign *X*_*i*_ to C_*l*_ for all *l*, 1≤ *l* ≤ *k*; 1≤*i*≤ *n* as Eq. (10)

11: Output Results

### Optimization of the problem *P*

In optimizing the problem *P*, the *k*-AMH algorithm uses a maximization process instead of the minimization process imposed by the *k*-Mode-type algorithms. This process is formalized in the *k*-AMH algorithm as follows.

Step 1 **-** Choose an Approximate Modal Haplotype, ***H***^***(t)***^***∈ X***. Calculate ***P(Á)***; Set ***t=1***

Step 2 - Choose ***X***^***(t+1)***^ such that ***P(Á)***^***t+1***^ is maximized; Replace ***H***^***1***^ by ***X***^***(t+1)***^

Step 3 **-** Set ***t=t+1*;** Stop when ***t=n***; otherwise go to Step 2.

*Note:*n is the number of objects*

The convergence of the algorithm is proven as *P*_*1*_ and *P*_*2*_ are maximized accordingly. The function *P(Á)* incorporates the *P(W, H)* function imposed by the Fuzzy *k*-Modes algorithm, where *W* is a partition matrix and *H* is the approximate modal haplotype that defines the center of a cluster. Thus, *P*_*1*_ and *P*_*2*_ are solved by Theorems 1 and 2, respectively.

**Theorem 1** – Let *Ĥ* be fixed. *P(W, Ĥ)* is maximized if and only if

Wli∝={1,If,Xi=Hi0,If,Xi=Hz,z≠l1∑z=1kdystrXi,HldystrXiHz1∝−1,IfHi≠XjandXi≠Hz,1≤z≤k∝

Proof

Let *X= {X*_*1*_*,X*_*2*_*,.,X*_*n*_*}* be a set of *n* Y-STR categorical objects and *H= {H*_*1*_*,H*_*2*_*,.,H*_*k*_} be a set of centroids (Approximate Modal Haplotypes) for *k* clusters. Suppose that *P= {P*_*1*_*,P*_*2*_*,.,P*_*k*_} is a set of dissimilarity measures based on *d*_*ystr*_(*X*_*i*,_*H*_*l*_), as described in Eqs. (5) and subject to (5a), ∀ *i* and *l* 1  ≤  *i*  ≤  *n;* 1  ≤  *l*  ≤  *k*

**Definition 1** - For *X*_*i*_  =  *H*_*l*_ and *X*_*i*_  =  *H*_*z*_, where *z*  ≠  *l*, the membership value for all *i* is

$$w_{\mathrm{li}}^{\propto }\;=\;\{{\left(\begin{array}{c} 1,\;if\;X_{i}\;=\;H_{l}\\ 0,\;if\;X_{i}\;=\;H_{z},\;z\;\neq \;l \end{array},\;\right)}^{\propto }$$

For any *P* that is obtained from *d*_*ystr*_(*X*_*i*,_*H*_*l*_) where *X*_*i*_  =  *H*_*l*_, the maximum value of *w*_*li*_^∝^ is 1 and *X*_*i*_  =  *H*_*z*_, *z*  ≠  *l* the value of *w*_*li*_^∝^ is 0. Therefore, because *H*_*l*_ is fixed, *w*_*li*_^∝^ is maximized.

**Definition 2** – For the case of *H*_*i*_  ≠  *X*_*i*_*and X*_*i*_ ≠ *H*_*z*_,   ∀  *z*,  1  ≤  *z*  ≤  *k*, the membership value for all *i* is

wli∝={1∑z=1kdystrXi,H^ldystrXi,H^z1∝−1∝

Suppose that *p*_*li*_ ∈ *P* is the minimum value, we write as

wli∝={1∑z=1kPliPzi1∝−1∝where1≤lk;1≤zk

={1PliP1i1∝−1+PliP2i1∝−1+PliPzi1∝−1+PliPki1∝−1∝

Therefore,

=PliPli1∝−1=1>=PliPzi1∝−1,

where *z* ≠ *l*

Thus, ∑z=1kPlizi1∝−1<∑z=1kPtiPzi1∝−1 where 

*t* ≠ *l* and ∀  *z and t*,  1  ≤  *z*  ≤  *k*;  1  ≤  *t*  ≤  *k* It follows that

wli∝={1∑z=1kPliPzi1∝−1∝>1∑z=1kPtiPzi1∝−1∝

where *t* ≠ *l*

Therefore, based on definitions 1 and 2, *w*_*li*_^∝^ is maximal. Because *Ĥ* is fixed, $P\left(W,\hat{H}\right)$ is maximized.

**Theorem 2** – Let *h*_*l*_ ∈ *X* be the initial center of a cluster for 1 *≤ l ≤ k*. *h*_*l*_ is replaced by *x*_*i*_ as the Approximate Modal Haplotype if and only if

$$P{\left(À\right)}^{s}>\;P{\left(À\right)}^{t};s\;\neq \;t;\;\forall t,\;1\;\leq \;t\;\leq \;\left(n-k\right)\text{.}$$

Proof

Let *D= {D*_*1*_*,D*_*2*_*,.,D*_*k*_} be a set of dominant weighting values. For any maximum value of *w*_*li*_^∝^ as proved by Theorem 1, we assign an optimum value of 1.0 as a dominant weighting value, otherwise 0.5 as described in Eq, (9) and subject to Eqs. (9a), (9b) and (9c). We write

$$P\left(A\right)=\sum_{l=1}^{k}\sum_{i=1}^{n}A_{\mathrm{li}}$$

$$=\sum_{l=1}^{k}\sum_{i=1}^{n}W_{\mathrm{li}}^{\alpha }D_{\mathrm{li}}$$

Because *w*_*li*_^∝^ and *D*_*li*_ are non-negative, the product *W*_*li*_^∝^*D*_*li*_ must be maximal. It follows that the sum of all quantities ∑ _*l* = 1_^*k*^ ∑ _*i* = 1_^*n*^*Á*_*li*_ is also maximal. Hence, the result follows.

### Y-STR Datasets

The Y-STR data were mostly obtained from a database called worldfamilies.net [30]. The first, second, and third datasets represent Y-STR data for haplogroup applications, whereas the fourth, fifth, and sixth datasets represent Y-STR data for Y-surname applications. All datasets were filtered for standardization on 25 similar attributes (25 markers). The chosen markers include DYS393, DYS390, DYS19 (394), DYS391, DYS385a, DYS385b, DYS426, DYS388, DYS439, DYS389I, DYS392, DYS389II, DYS458, DYS459a, DYS459b, DYS455, DYS454, DYS447, DYS437, DYS448, DYS449, DYS464a, DYS464b, DYS464c, and DYS464b. These markers are more than sufficient for determining a genetic connection between two people. According to Fitzpatrick [31], 12 markers (Y-DNA12 test) are already sufficient to determine who does or does not have a relationship to the core group of a family.

All datasets were retrieved from the respective websites in April 2010, and can be described as follows:

1) The first dataset consists of 751 objects of the Y-STR haplogroup belonging to the Ireland yDNA project [32]. The data contain only 5 haplogroups, namely E (24), G (20), L (200), J (32), and R (475). Thus, *k* = 5.

2) The second dataset consists of 267 objects of the Y-STR haplogroup obtained from the Finland DNA Project [33]. The data are composed of only 4 haplogroups: L (92), J (6), N (141), and R (28). Thus, *k* = 4.

3) The third dataset consists of 263 objects obtained from the Y-haplogroup project [34]. The data contain Groups G (37), N (68), and T (158). Thus, *k* = 3.

4) The fourth dataset consists of 236 objects combining four surnames: Donald [35], Flannery [36], Mumma [37], and William [38]. Thus, *k* = 4.

5) The fifth dataset consists of 112 objects belonging to the Philips DNA Project [39]. The data consist of eight family groups: Group 2 (30), Group 4 (8), Group 5 (10), Group 8 (18), Group 10 (17), Group 16 (10), Group 17 (12), and Group 29 (7). Thus, *k* = 8.

6) The sixth dataset consists of 112 objects belonging to the Brown Surname Project [40]. The data consist of 14 family groups: Group 2 (9), Group 10 (17), Group 15 (6), Group 18 (6), Group 20 (7), Group 23 (8), Group 26 (8), Group 28 (8), Group 34 (7), Group 44 (6), Group 35 (7), Group 46 (7), Group 49 (10), and Group 91 (6). Thus, *k* = 14.

The values in parentheses indicate the number of objects belonging to that particular group. Datasets 1–3 represent Y-STR haplogroups and datasets 4–6 represent Y-STR surnames.

## Results and discussion

The following results compare the performance of the *k*-AMH algorithm with eight other partitional algorithms: the *k*-Modes algorithm [25], *k*-Modes with RVF [21,22,41], *k*-Modes with UAVM [21], *k*-Modes with Hybrid 1 [21], *k*-Modes with Hybrid 2 [21], the Fuzzy *k*-Modes algorithm [24], the *k*-Population algorithm [23], and the New Fuzzy *k*-Modes algorithm [20].

Our analysis was based on the average accuracy scores obtained from 100 runs for each algorithm and dataset. During the experiments, the objects in the datasets were randomly reordered from the preceding run. The misclassification matrix proposed by Huang [25] was used to obtain the clustering accuracy scores for evaluating the performance of each algorithm. The clustering accuracy *r* defined by Huang [25] is given by Eq. (11):

(11) $$r=\frac{\sum_{i=1}^{k}a_{i}}{n}$$

where *k* is the number of clusters, *a*_*i*_ is the number of instances occurring in both cluster *i* and its corresponding haplogroup or surname, and *n* is the number of instances in the dataset.

### Clustering performance

Table 2 shows the clustering accuracy scores for all datasets (boldface indicates the highest clustering accuracy). Based on these results, the performance of the *k*-AMH algorithm was very promising. Out of six datasets, our algorithm obtained the highest clustering accuracy scores for datasets 1, 2, 4, 5, and 6. In fact, the algorithm also achieved the optimal clustering accuracy for two datasets (4 and 5). However, for dataset 3, the results show that the accuracy of the *k*-AMH algorithm was 0.01 lower than that of the *k*-Population algorithm. A statistical *t*-test was carried out for further verification. This indicated that *t*(101.39) = 0.65, and *p* = 0.51. Thus, there was no significant difference at the 5% level between the accuracy score of our *k*-AMH algorithm and the *k*-Population algorithm. This means that both algorithms displayed an equal performance for this dataset.

**Table 2:** Clustering accuracy scores for all datasets

**ALGORITHM**	**DATASET**					
	**1**	**2**	**3**	**4**	**5**	**6**
*k*-Modes	0.70	0.79	0.84	0.84	0.74	0.62
*k*-Modes-RVF	0.79	0.83	0.87	0.78	0.87	0.72
*k*-Modes-UAVM	0.65	0.75	0.83	0.87	0.56	0.54
*k*-Modes-Hybrid 1	0.67	0.81	0.85	0.77	0.80	0.64
*k*-Modes-Hybrid 2	0.56	0.82	0.83	0.79	0.81	0.70
Fuzzy *k*-Modes	0.56	0.74	0.74	0.97	0.76	0.66
*k*-Population	0.80	0.90	**0.97**	1.00	0.97	0.84
New Fuzzy *k*-Modes	0.71	0.84	0.77	1.00	0.77	0.69
***k*-AMH**	**0.83**	**0.93**	0.96	**1.00**	**1.00**	**0.87**

During the experiments, the *k*-AMH algorithm did not encounter any difficulties. However, the Fuzzy *k*-Modes and the New Fuzzy *k*-Modes algorithms faced problems with datasets 1, 5, and 6. For dataset 1, the problem was caused by the extreme number of objects in Class *R* (475), which covered about 63% of the total objects. Further, for datasets 5 and 6, the problem was caused by many similar objects in a larger number of classes. In particular, both algorithms faced the problem *P*_*2*_ caused by the initial centroid selections. Note also that the results for both algorithms were based on the diverse method, an initial centroid selection proposed by Huang [25].

For an overall comparison, Table 3 shows the results of all Y-STR datasets. It clearly indicates that the *k*-AMH algorithm obtained the highest accuracy score of 0.93. The closest score of 0.91 belongs to the *k*-Population algorithm. Furthermore, the *k*-AMH algorithm also recorded the best results in terms of standard deviation (0.07), the lower bound (0.93), the upper bound (0.94), and the minimum accuracy score (0.79).

**Table 3:** Clustering accuracy scores for all Y-STR datasets

	**N**	**Mean**	**Std. Dev.**	**95% Confidence Interval for Mean**		**Min**	**Max**
				**Lower Bound**	**Upper Bound**		
*k*-Mode	600	0.76	0.13	0.75	0.77	0.45	1.00
*k*-Mode-RVF	600	0.81	0.11	0.80	0.82	0.56	1.00
*k*-Mode-UAVM	600	0.70	0.17	0.69	0.71	0.38	1.00
*k*-Mode-Hybrid 1	600	0.76	0.13	0.75	0.77	0.38	1.00
*k*-Mode-Hybrid 2	600	0.75	0.14	0.74	0.76	0.45	1.00
Fuzzy *k*-Mode	600	0.74	0.16	0.73	0.75	0.32	1.00
*k*-Population	600	0.91	0.09	0.91	0.92	0.59	1.00
New Fuzzy *k*-Mode	600	0.80	0.13	0.79	0.81	0.44	1.00
***k*-AMH**	**600**	**0.93**	**0.07**	**0.93**	**0.94**	**0.79**	**1.00**

For further verification, a one-way ANOVA test was carried out. This indicated that the assumption of homogeneity of variance was violated; therefore, the Welch *F*-ratio is reported. There was a significant variance in the clustering accuracy scores among the nine algorithms, in which *F*(8, 2230) = 378, *p* < 0.001, and *ω*^*2*^ = 0.25. Thus, the Games–Howell procedure was used for a multiple comparison among the nine algorithms. Table 4 shows the result of this comparison with regard to the *k*-AMH algorithm against the other eight algorithms. At the 5% level of significance, it is clearly shown that the *k*-AMH algorithm (*M* = 0.93, 95% CI [0.93, 0.94]) differed from the other eight algorithms (all *P* values < 0.001). Thus, the performance of *k*-AMH algorithm exhibited a very significant difference compared to the other algorithms.

**Table 4:** **Multiple comparisons for the *k*-AMH algorithm**

**Accuracy Games–Howell**						
**(I) Algorithm**	**(J) Algorithm**	**Mean Diff. (I-J)**	**Std. Error**	***p*-value**	**95% Confidence Interval**	
					**Lower Bound**	**Upper Bound**
*k*-AMH	*k*-Mode	0.17^*^	0.01	**< 0.00001**	0.16	0.19
	*k*-Mode-RVF	0.12^*^	0.01	**< 0.00001**	0.11	0.14
	*k*-Mode-UAVM	0.23^*^	0.01	**< 0.00001**	0.21	0.25
	*k*-Mode-Hybrid 1	0.17^*^	0.01	**< 0.00001**	0.16	0.19
	*k*-Mode-Hybrid 2	0.18^*^	0.01	**< 0.00001**	0.16	0.20
	Fuzzy *k*-Mode	0.19^*^	0.01	**< 0.00001**	0.17	0.21
	*k*-Population	0.02^*^	0.00	**0.00271**	0.01	0.03
	New Fuzzy *k*-Modes	0.13^*^	0.01	**< 0.00001**	0.12	0.15

*Note: p < 0.05.

### Efficiency

We now consider the time efficiency of the *k*-AMH algorithm. The computational cost of the algorithm depends on the nested loop for *k(n-k)*, where *k* is the number of clusters and *n* is the number of data required to obtain the cost function, *P(À)*. The function *P(À)* involves the number of attributes *m* in calculating the distances and the membership values for its partition matrix *w*_*li*_. Thus, the overall time complexity is *O*(*km*(*n-k*)). However, the time efficiency of the *k*-AMH algorithm will not reach *O(n*^*2*^*)* because the value of *k* in the outer loop will not become equivalent to the value of *n-k* in the inner loop. See pseudo-code for a detailed implementation of these loops.

**Figure 5:**
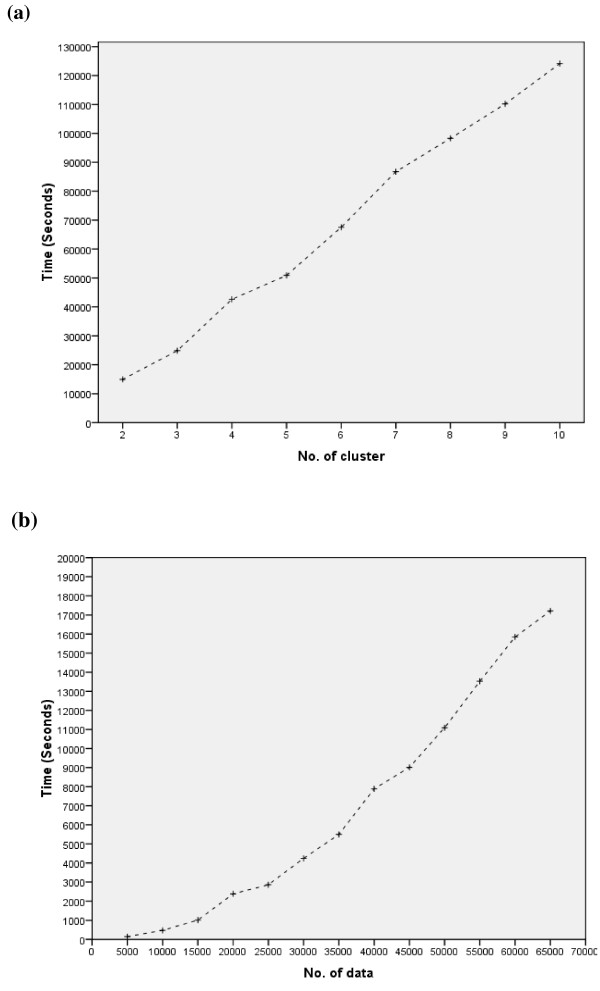
**Scalability Testing. a **Execution time to cluster 65,000 data into different numbers of clusters. **b **Execution time to cluster a different number of data into three clusters.

A scalability test was also carried out for the *k*-AMH algorithm. These experiments were based on a dataset called Connect [42]. This dataset consisted of 65,000 data, 42 attributes, and three classes. Two scalability tests were conducted: (a) scalability against the number of objects, when the number of clusters was three, and (b) scalability against the number of clusters, when the number of objects was 65,000. The test was performed on a personal computer with an Intel® Core™ 2 DUO Processor with 2.93 GHz and 2.00 GB memory. Figure 5(a) and (b) illustrate the results of the tests. In conclusion, the runtime of the *k*-AMH algorithm increased linearly with the number of clusters and data.

## Conclusions

Our experimental results indicate that the performance of the proposed *k*-AMH algorithm for partitioning Y-STR data was significantly better than that of the other algorithms. Our algorithm handled all problems, as described previously, and was not too sensitive to *P*_*0*_, the initial centroid selection, even though the datasets contained a lot of similar objects. Moreover, the concept of *P*_*2*_ in using the object (the data itself) as the approximate center of a cluster has significantly improved the overall performance of the algorithm. In fact, our algorithm is the most consistent of those tested because the difference between the minimum and maximum scores is smaller. The *k*-AMH algorithm always produces the highest minimum score for each dataset. In conclusion, the *k*-AMH algorithm is an efficient method of partitioning Y-STR categorical data.

## Competing interests

The authors declare that they have no competing interests.

## Authors' contributions

AS carried out the algorithm development and experiments. ZAB verified the algorithm and the results. MNI verified the Y-STR data and also the results. All authors read and approved the final manuscript.

## References

[B1] Kayser M Kittler R Erler A Hedman M Lee AC Mohyuddin A Mehdi SQ Rosser Z Stoneking M Jobling MA Sajantila A Tyler-Smith C A comprehensive survey of human Y-chromosomal microsatellites Am J Hum Genet 2004 74 6 1183 1197 10.1086/421531 15195656 PMC1182082

[B2] Perego UA Turner A Ekins JE Woodward SR The science of molecular genealogy National Genealogical Society Quarterly 2005 93 4 245 259

[B3] Perego UA The power of DNA: Discovering lost and hidden relationships 2005 Oslo: World Library and Information Congress: 71st IFLA General Conference and Council Oslo

[B4] Hutchison LAD Myres NM Woodward S Growing the family tree: The power of DNA in reconstructing family relationships Proceedings of the First Symposium on Bioinformatics and Biotechnology (BIOT-04) 2004 1 42 49

[B5] Dekairelle AF Hoste B Application of a Y-STR-pentaplex PCR (DYS19, DYS389I and II, DYS390 and DYS393) to sexual assault cases Forensic Sci Int 2001 118 122 125 10.1016/S0379-0738(00)00481-3 11311823

[B6] Rolf B Keil W Brinkmann B Roewer L Fimmers R Paternity testing using Y-STR haplotypes: Assigning a probability for paternity in cases of mutations Int J Legal Med 2001 115 12 15 10.1007/s004140000201 11599763

[B7] Dettlaff-Kakol A Pawlowski R First polish DNA “manhunt” - an application of Y-chromosome STRs Int J Legal Med 2002 116 289 291 12376840 10.1007/s00414-002-0320-0

[B8] Stix G Traces of the distant past Sci Am 2008 299 56 63 10.1038/scientificamerican0708-56 18623965

[B9] Gerstenberger J Hummel S Schultes T Häck B Herrmann B Reconstruction of a historical genealogy by means of STR analysis and Y-haplotyping of ancient DNA Eur J Hum Genet 1999 7 469 477 10.1038/sj.ejhg.5200322 10352937

[B10] International Society of Genetic Genealogyhttp://www.isogg.org

[B11] The Y Chromosome Consortiumhttp://ycc.biosci.arizona.edu

[B12] Schlecht J Kaplan ME Barnard K Karafet T Hammer MF Merchant NC Machine-learning approaches for classifying haplogroup from Y chromosome STR data PLoS Comput Biol 2008 4 6 e1000093 10.1371/journal.pcbi.1000093 18551166 PMC2396484

[B13] Seman A Abu Bakar Z Mohd Sapawi A Centre-based clustering for Y-Short Tandem Repeats (Y-STR) as Numerical and Categorical data Proc. 2010 Int. Conf. on Information Retrieval and Knowledge Management (CAMP’10) 2010 1 28 33 Shah Alam, Malaysia

[B14] Seman A Abu Bakar Z Mohd Sapawi A Centre-Based Hard and Soft Clustering Approaches for Y-STR Data Journal of Genetic Genealogy 2010 6 1 1 9 Available online: http://www.jogg.info

[B15] Seman A Abu Bakar Z Mohd Sapawi A Attribute Value Weighting in K-Modes Clustering for Y-Short Tandem Repeats (Y-STR) Surname Proc. of Int. Symposium on Information Technology 2010 (ITsim’10) 2010 3 1531 1536 Kuala Lumpur, Malaysia

[B16] Seman A Abu Bakar Z Mohd Sapawi A Hard and Soft Updating Centroids for Clustering Y-Short Tandem Repeats (Y-STR) Data Proc. 2010 IEEE Conference on Open Systems (ICOS 2010) 2010 1 6 11 Kuala Lumpur, Malaysia

[B17] Seman A Abu Bakar Z Mohd Sapawi A Modeling Centre-based Hard and Soft Clustering for Y Chromosome Short Tandem Repeats (Y‐STR) Data Proc. 2010 International Conference on Science and Social Research (CSSR 2010) 2010 1 73 78 Kuala Lumpur, Malaysia

[B18] Seman A Abu Bakar Z Mohd Sapawi A Centre-based Hard Clustering Algorithm for Y-STR Data Malaysia Journal of Computing 2010 1 62 73

[B19] Seman A Abu Bakar Z Isa MN Evaluation of k-Mode-type Algorithms for Clustering Y-Short Tandem Repeats Journal of Trends in Bioinformatics 2012 5 2 47 52 10.3923/tb.2012.47.52

[B20] Ng M Jing L A new fuzzy k-modes clustering algorithm for categorical data International Journal of Granular Computing, Rough Sets and Intelligent Systems 2009 1 1 105 119 10.1504/IJGCRSIS.2009.026727

[B21] He Z Xu X Deng S Attribute value weighting in k-Modes clustering 2007 Ithaca, NY, USA: Cornell University Library, Cornell University 1 15 available online: http://arxiv.org/abs/cs/0701013v1

[B22] Ng MK Junjie M Joshua L Huang Z He Z On the impact of dissimilarity measure in k-modes clustering algorithm IEEE Trans Pattern Anal Mach Intell 2007 29 3 503 507 17224620 10.1109/TPAMI.2007.53

[B23] Kim DW Lee YK Lee D Lee KH k-Populations algorithm for clustering categorical data Pattern Recogn 2005 38 1131 1134 10.1016/j.patcog.2004.11.017

[B24] Huang Z Ng M A Fuzzy k-Modes algorithm for clustering categorical data IEEE Trans Fuzzy Syst 1999 7 4 446 452 10.1109/91.784206

[B25] Huang Z Extensions to the k-Means algorithm for clustering large datasets with categorical values Data Min Knowl Discov 1998 2 283 304 10.1023/A:1009769707641

[B26] MacQueen JB Some methods for classification and analysis of multivariate observations The 5th Berkeley Symposium on Mathematical Statistics and Probability 1967 1 281 297

[B27] Ralambondrainy H A conceptual version of the k-Means algorithm Pattern Recogn Lett 1995 16 1147 1157 10.1016/0167-8655(95)00075-R

[B28] Bobrowski L Bezdek JC c-Means clustering with the l1 and l∞ norms IEEE Trans Syst Man Cybern 1989 21 3 545 554

[B29] Salim SZ Ismail MA k-Means-type algorithms: A generalized convergence theorem and characterization of local optimality IEEE Trans Pattern Anal Mach Intell 1984 6 81 87 21869168 10.1109/tpami.1984.4767478

[B30] WorldFamilies.nethttp://www.worldfamilies.net

[B31] Fitzpatrick C Forensic genealogy 2005 Fountain Valley: Cal.: Rice Book Press

[B32] Ireland yDNA project http://www.familytreedna.com/public/IrelandHeritage/

[B33] Finland DNA Project http://www.familytreedna.com/public/Finland/

[B34] Y-Haplogroup projecthttp://www.worldfamilies.net/yhapprojects/

[B35] Clan Donald Genealogy Projecthttp://dna-project.clan-donald-usa.org

[B36] Flannery Clanhttp://www.flanneryclan.ie

[B37] Doug and Joan Mumma’s Home Pagehttp://www.mumma.org

[B38] Williams Genealogyhttp://williams.genealogy.fm

[B39] Phillips DNA Project. http://www.phillipsdnaproject.com

[B40] Brown Genealogy Societyhttp://brownsociety.org

[B41] San OM Huynh V Nakamori Y An alternative extension of the K-Means Algorithm for clustering categorical data IJAMCS 2004 14 2 241 247

[B42] Blake CL Merz CJ UCI repository of machine learning database 1989

